# Augmented and Virtual Reality for Improving Safety in Railway Infrastructure Monitoring and Maintenance

**DOI:** 10.3390/s25123772

**Published:** 2025-06-17

**Authors:** Marina Ricci, Nicola Mosca, Maria Di Summa

**Affiliations:** Institute of Intelligent Industrial Technologies and Systems for Advanced Manufacturing (STIIMA), National Research Council of Italy, via Amendola 122/D-I, 70126 Bari, Italy; nicola.mosca@stiima.cnr.it (N.M.); maria.disumma@stiima.cnr.it (M.D.S.)

**Keywords:** augmented and virtual reality, maintenance, human–computer interaction, interaction design, user interface

## Abstract

The highly demanding safety standards adopted in the railway context imply that cutting-edge technologies must limit accidents. This paper presents the human-centered outcomes of the VRAIL project, an industrial research project aiming to use enabling technologies and develop methodologies for operators directly involved in infrastructure management in the railway field. Developing integrated monitoring systems and applications that exploit Augmented Reality (AR) and Virtual Reality (VR) becomes crucial to support the awareness of planning and maintenance operators required to comply with high-quality standards. This paper addresses the abovementioned issue by proposing the development of two different prototype applications in both AR and VR for railway infrastructure data management. These environments will provide the planning operator with a complete platform to explore, use to plan maintenance interventions, and gather detailed reports to improve the overall safety of the railway line effectively.

## 1. Introduction

Nowadays, rail transport plays a crucial role in transporting passengers and goods. In this field, safety is a key aspect that requires substantial resources and attracts continued interest from research and industry [1]. Several companies, often on a global scale, operate in this area and are developing hardware/software solutions and ad hoc consulting services. Although investment in railway safety has grown exponentially in recent years, there is still room for improvement and risk mitigation [2]. Addressing this problem involves upgrading technological tools to assist human operators in making informed decisions, with Augmented and Virtual Reality (AR, VR) serving as key enabling technologies [3].

Literature has widely proven that both technologies can significantly enhance the effectiveness of training programs, promote a deeper understanding of complex procedures, and provide safer, cost-effective solutions for high-risk sectors like the railway industry. By creating immersive scenarios that closely replicate real-world environments, VR allows rail personnel to practice emergency procedures, maintenance tasks, and operational routines without endangering lives or disrupting actual service. At the same time, AR overlays critical information—such as track layouts, equipment status, and safety alerts—directly onto the physical environment, supporting on-site decision-making and reducing the margin of human error. When applied to railway safety, the integration of VR offers key advantages, such as allowing trainees to repeatedly experience realistic, high-risk conditions, like signaling malfunctions or unexpected obstacles on the tracks, in a controlled environment, thereby building confidence and know-how essential for real emergencies. It also reduces operational costs by minimizing the need to halt service or deploy costly equipment for traditional on-track training exercises. Moreover, AR-based interfaces can provide frontline workers immediate access to diagnostic tools, step-by-step repair instructions, or hazard warnings in their field of view, enhancing both safety and efficiency. Data gathered during VR and AR sessions offers valuable insights for continuous performance monitoring, helping identify knowledge gaps and refine training strategies. Therefore, embedding VR and AR into railway safety protocols bolsters personnel knowledge and fosters a culture of innovation and proactive risk management.

This paper aims to address the issue by developing two prototypes of applications that exploit the human-centered design (HCD) approach—one integrating VR and one AR—to use and manage railway infrastructure data. More broadly, the goal is to create a modular, integrated system that combines VR and AR components to support railway monitoring and maintenance activities in the field directly. As demonstrated in our previous study [4], where we introduced the conceptual framework for integrating deep learning-based anomaly detection with extended reality (XR) technologies, this work moves a step forward by exploring the design and implementation of the individual VR and AR modules. These modules are intended to serve as functional components of a comprehensive XR-based system that enhances safety, decision making, and operational efficiency in the railway sector.

Another previous study concerned an unsupervised deep learning approach for detecting missing bolts on railway tracks using 3D imaging data [5]. The method was based on training an autoencoder on depth maps of safe rail segments to identify structural anomalies. The approach proved particularly effective in scenarios with limited labeled data, achieving high recall and no false negatives. This work laid the foundation for our current research, which expands from anomaly detection to immersive, operator-oriented AR and VR systems for infrastructure monitoring and decision support.

The remainder of this paper is structured into three sections. The first describes the state of the art related to VR and AR railway infrastructure monitoring activities to enhance safety. The second describes the methodology for designing the applications. The third reports on the application specifications and functions. Lastly, we will report our conclusions and future work.

## 2. Background

Monitoring and maintaining railway infrastructure is critical for ensuring reliable, safe transportation across extensive networks. However, these networks’ inherent complexity and scale present ongoing challenges in detecting, diagnosing, and resolving potential issues before they escalate into safety hazards. Over the past decade, emerging digital technologies, specifically VR and AR, have shown promise in addressing these challenges by providing immersive, interactive environments that can enhance real-time data visualization, remote collaboration, and worker training.

Despite growing interest, the literature reveals a fragmented landscape in which VR and AR applications are often addressed in isolation, with limited integration into comprehensive, data-driven maintenance and diagnostic workflows. This fragmentation indicates a significant research gap: the lack of unified, operationally viable frameworks that combine immersive visualization with intelligent data analysis tools for infrastructure monitoring. Addressing this gap is essential for unlocking the full potential of VR and AR in transforming railway safety practices.

This section offers a comprehensive overview of the current landscape of VR and AR applications in railway infrastructure monitoring, highlighting both their technical foundations and the specific safety improvements these tools can enable.

### 2.1. Virtual Reality

The literature on VR in the railway sector highlights how this technology offers significant potential, particularly in training and simulating complex operational scenarios [6]. Many studies have shown that developing immersive and interactive environments enables personnel to practice under conditions as close to reality as possible, addressing emergencies, technical malfunctions, and extraordinary maintenance operations without exposing people and infrastructure to actual risks [7,8].

Several studies have focused on creating advanced simulators that replicate signaling systems, station layouts, and interactions with railway vehicles, demonstrating a significant improvement in operators’ ability to manage critical procedures correctly [9].

In the study by Liu et al. [10], VR technology is employed to control the learning environment of Convolutional Neural Networks (CNNs) for railway applications. This approach allows for the creation of controlled, immersive environments where CNNs could be trained effectively, demonstrating the potential of VR in enhancing machine learning processes within the railway sector.

Further advancements in 3D visualization for railway infrastructure inspection are discussed by McDonald et al. [11]. Their comprehensive review focuses on the current state of data acquisition and information conveyance schemes for Railway Tunnel SubSurface Inspection. The study highlights the importance of effective visualization frameworks in detecting and characterizing concealed features within railway tunnels, emphasizing the role of 3D visualization in improving maintenance strategies.

These studies underscore the transformative potential of integrating VR, CNNs, and 3D visualization technologies in railway systems. By leveraging these advanced tools, the railway industry can significantly improve training efficacy, infrastructure inspection, and overall operational safety. Overall, the research findings suggest that adopting VR tools in the railway sector can lead to increased safety, reduced training costs, and an overall improvement in the quality and effectiveness of maintenance operations.

However, these contributions treat VR applications as standalone solutions, without embedding them in integrated decision-making frameworks supported by real-time diagnostic data. This disconnection between immersive technology and actionable data insight points to an underexplored area that our research aims to bridge.

### 2.2. Augmented Reality

Recent literature on the AR application in the railway sector highlights how this technology can play a strategic role both in infrastructure inspection and maintenance activities and in personnel training and upskilling [12]. Some studies focus on using AR devices to visualize real-time data on the condition of tracks and structural components, enabling technicians to detect defects or potential malfunctions quickly [13]. Other research has explored the integration of sensors and data analytics platforms to provide insights into possible anomalies, displaying contextual suggestions and instructions directly within the operator’s field of view [3]. From a training perspective, several authors emphasize that AR enhances immersive learning by offering operational simulations that accurately replicate the environmental complexity and critical challenges typical of the railway sector [14].

Despite the strategic significance of the railway sector, the adoption of AR remains limited, with most applications primarily focused on maintenance. For instance, Azpiazu et al. [15] introduce a remote support system in which a worker, using AR glasses, could communicate directly with a specialist who provides visual annotations, such as arrows or symbols, in the worker’s field of view, thereby enhancing maintenance efficiency and reducing costs. Similarly, Kwon et al. [16] developed an AR-based system for maintenance and training on the Axle-Mounted Disc Brake System, utilizing handheld devices such as tablets and smartphones. Their findings demonstrate a 34% improvement in efficiency and a significant reduction in errors.

Beyond maintenance, AR has also been employed to inspect freight car routes carrying out-of-gauge cargo [17]. An AR-based interference detection system is used to identify potential collisions between the train and surrounding infrastructure, facilitating optimal route selection. Compared to conventional methods, this approach proves to be more cost-effective and highly precise.

An effective AR system should, first and foremost, ensure process continuity, as any interruptions or inconsistencies could compromise functionality. Additionally, the system must be intuitive and accessible, particularly for users with limited experience in AR technology. To achieve this, the interface should be user-friendly, minimizing functional inconsistencies and actively involving end users throughout development. These considerations have been fundamental in designing the AR system, described in detail in the following sections.

Yet, as in the case of VR, current AR research often stops at proof-of-concept or small-scale implementations, without fully exploiting diagnostic data or integrating Artificial Intelligence (AI) -driven insights. This suggests an urgent need for frameworks that employ AR as a visualization layer and tightly couple it with smart analytics to support informed, in-field decision making. Our research builds directly on this premise.

## 3. Methods

This study addresses critical research problems currently limiting the effectiveness of railway infrastructure monitoring. Although modern diagnostic systems generate vast amounts of data, this information is often underutilized due to fragmented access, limited visualization tools, and insufficient integration with maintenance workflows. Operators still rely on 2D interfaces and manual interpretations, resulting in delays, inefficiencies, and increased safety risks. Moreover, existing VR and AR tools are typically developed in isolation, without integration into scalable, data-driven decision-making systems. To overcome these challenges, our research is guided by the following objectives:

- To design and implement an integrated VR/AR system supporting immersive training and real-time, in-field decision making for railway infrastructure maintenance.
- To connect diagnostic data and intelligent anomaly detection (via AI and machine learning) with immersive visual interfaces to enhance situational awareness and reduce operator subjectivity.
- To apply a HCD methodology to ensure usability and accessibility of the developed tools across different user roles.
- To evaluate the effectiveness of the proposed system in improving the accuracy, efficiency, and traceability of inspection and maintenance tasks.

While this paper focuses primarily on technical development and early-stage usability assessment, preliminary qualitative feedback was collected through iterative lab sessions with end users.

The paper is framed within the VRAIL project, an industrial research project aiming to exploit enabling technologies and develop methodologies for operators directly involved in infrastructure management, including infrastructure managers, managers, and maintenance workers. The idea of the project is to use tools based on non-traditional interaction approaches, such as visual technological solutions and communication technologies, including VR and AR, but also advanced data processing techniques, such as AI, machine learning, and deep learning.

This section builds on the abovementioned gaps by illustrating how our proposed solution integrates immersive technologies with intelligent data analysis to overcome the disconnect between diagnostic potential and operational implementation.

Indeed, there is still a considerable gap in the railway sector between the amount of diagnostic data available and its ability to provide benefits when consulted. The data that guides the operators’ decisions often remains limited to two-dimensional paper and mobile interfaces. We are considering using technologies such as VR and AR to bridge the gap between the vast amount of data available and the ability to benefit from it. The applications to be developed allow, on the one hand, to use and make available all the diagnostic data of the railway line acquired in the field by sophisticated measurement systems and, on the other hand, to return information, such as defects, measurement data, and insights deriving from sophisticated integrated processing.

**Figure 1:**
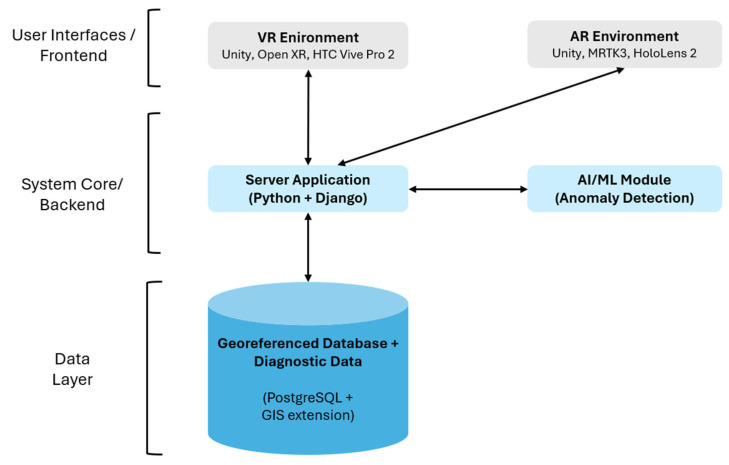
System architecture of the integrated VR/AR platform for railway monitoring and maintenance.

In our previous study related to the VRAIL research project [4], we proposed a framework by introducing a three-stage pipeline consisting of a VR Environment (VRE) for immersive visualization, an Anomaly Detector (AD) for automatic defect identification, and an AR Engine (ARE) for in-field decision support. The system is based on a client-server architecture (See Figure 1), where a server application and a georeferenced database serve as the central repository for information shared across various modules. Data exchange primarily relies on the JSON format. The server application is developed in Python using the Django framework. A georeferenced database, such as PostgreSQL with its Geographic Information System extension, is used for storing and querying maintenance-related assets. Managed assets include, for example, level crossings, rail switches, levers, and bolts. These assets are represented in the system as needed and visualized within VR and AR scenes, along with other virtual accessory objects, such as virtual totems (UI elements). A key aspect in achieving the project objectives was the recognition of physical assets, such as the levers located near rail switches. These elements are used in the AR system to identify potential defects that need further investigation on-site. Recognition of such landmarks was implemented using the Vuforia Engine [18], leveraging its model target recognition capabilities. The availability of an official plugin capable of running natively on the HoloLens 2 was critical for this task. It is important to note that, while components such as model target recognition and system architecture are essential to the project, they will not be further explored in this paper, which focuses primarily on the presentation aspects of the developed solution. Compared to traditional railway inspection methods, which often suffer from high costs and operator subjectivity, this integrated framework provides a more scalable, objective, and immersive approach to railway safety monitoring. This paper explores and presents the VR and AR systems designed and developed to achieve the research project objectives.

For designing the AR and VR applications, we exploit an HCD approach that, as reported in ISO13407:1999, guides the design of interactive systems development to make systems usable and useful by focusing on the users, their needs, and requirements, and by applying human factors/ergonomics, usability knowledge, and techniques [19,20].

The HCD approach consists of the following four iterative steps:

1. Identification of the context of the use and research on users.
2. Definition of user needs and requirements, and system requirements.
3. Develop design solutions that can meet the needs and requirements.
4. Evaluation of the design solution against requirements.

In our proposal, we integrate AR and VR into applications based on users’ active involvement to identify user needs, user and system requirements, their tasks, the context of use, and an appropriate allocation between users and the system of functions to be developed. Specifically, two prototypes (i.e., proof of concept) have been designed to use and manage railway infrastructure data through VR and AR. In both applications, the user interface (UI) was developed through a co-design process between our research team and the partner company, considering user needs, user requirements, and system requirements. Evaluation efforts focused on lab-based testing with expert users. Although these sessions were not focused on acquiring statistically significant metrics, they guided design iterations and provided insight into usability and system clarity. A structured experimental protocol—including benchmark tasks for both traditional and immersive systems—is currently under development and will form the basis of future evaluation studies. Although the UI was not tested in a full on-site deployment, iterative sessions were conducted in a lab setting with end users to refine interaction mechanisms progressively.

## 4. Results

This section presents the implemented AR and VR applications developed to enhance railway infrastructure monitoring. As our previous study [3] highlighted, the VR and AR modules (i.e., VRE and ARE) address well-defined and separate use cases. These applications were successfully designed and deployed, demonstrating their functionality in facilitating defect detection, inspection procedures, and operator support. The following subsections describe the implementation outcomes, highlighting their effectiveness and potential benefits in the railway sector.

### 4.1. VR Application

We developed the VR application using Unity 3D 2021 as the game engine, using the OpenXR plugin and XR Interaction Toolkit. The virtual scene was built using point clouds of a leading European company in railway diagnostics, acquired through Leica BLK360, which served as the case study environment for the field studies. The raw point cloud data were preprocessed and refined using CloudCompare, a specialized point cloud editing and analysis software.

It is important to note that a point cloud representation was preferred over polygonal meshes, as it was better suited to the project’s objectives and performance requirements. The preprocessing phase focused on removing portions of the environment that were not relevant to maintenance activities, thereby reducing computational complexity. Nonetheless, a polygonal mesh was derived from the point cloud terrain. The selected point cloud sections were decimated and then triangulated, resulting in a static mesh. This mesh was then used as a collider surface to enable teleportation operations within the virtual environment.

After necessary modifications, the processed point clouds were imported into Unity 3D, serving as the spatial foundation of the virtual environment, by using the Point Cloud Viewer and Tools plugin. The latter also provided an optimized shader for rendering the imported point meshes using the built-in pipeline [21]. Most virtual assets consisted of virtual totems, similar to those used in digital signage, and were modeled using Blender. These totems—world-space UI panels (or spatial UI panels) [22,23]—served as user guidance elements, particularly aimed at assisting first-time users. As with several other features of the VR and AR applications, their inclusion resulted from iterative development phases, informed by informal feedback from stakeholders.

Once the base scene was established, we incorporated virtual assets to be overlaid onto the point cloud model. These assets were explicitly configured to enable interaction with selected elements of the scene, ensuring a dynamic and engaging user experience. The primary objective was to create a realistic and interactive environment, allowing users to visualize the study scenario and engage with its key components. The virtual scene was rendered and experienced using the HTC Vive Pro 2 to enable immersive visualization and interaction. The Head-Mounted Display (HMD) positional tracking and hand controller inputs facilitated user navigation and real-time interaction with the augmented elements, enhancing the simulation’s realism, depth perception, and engagement.

Regarding interaction design, we adopted a user-centered approach informed by early-stage feedback from end users. Our primary objective was to simplify the UI and promote intuitive interactions tailored to the specific needs identified by our target audience. We implemented a teleportation-based locomotion system to navigate effectively within expansive virtual spaces [24,25]. This mechanism, activated by pointing the controller and pressing a trigger, reduces physical strain and disorientation while ensuring consistency, as it also serves as a means for interacting with panels and selecting options.

To further streamline the user experience, we consciously minimized the UI elements and buttons, significantly lowering cognitive load. Instructional content and task-specific guidance were delivered via strategically positioned virtual totems. These totems functioned as centralized, visually clear, and contextual reference points, effectively guiding users through the bolt inspection and classification processes.

The core interaction principles—spatial clarity, minimalistic gestures, and progressive disclosure—were chosen to enhance usability and immersion, benefiting users unfamiliar with VR technology.

In the UI design, virtual totems were leveraged as intuitive and consistent hubs to convey textual and visual instructions. By strategically placing these totems throughout the virtual environment, users were guided step by step through the simulation, focusing on task execution without distraction from complex or scattered UI components. This approach preserved immersion and provided a reliable navigation, decision making, and action confirmation reference point.

In particular, the target users of the VR module are maintenance mission planners. Their primary objective is to schedule and dispatch repair teams by coordinating maintenance operations. The output of the planning process consists of detailed instructions and logistical arrangements. Planners must coordinate with the railway service operator to ensure that train services are interrupted appropriately, allowing the repair team to reach the site, carry out maintenance tasks, and produce comprehensive pre- and post-intervention reports. They are also responsible for providing the team with accurate guidance and equipment. A VR solution supports the planning team by enabling effective remote analysis and preparation, eliminating the need for prior physical inspections of the site. Using a point cloud as the primary method for presenting the acquired scene allowed maintenance planners to experience the environment in a form that closely preserved its original and unaltered appearance.

**Figure 2:**
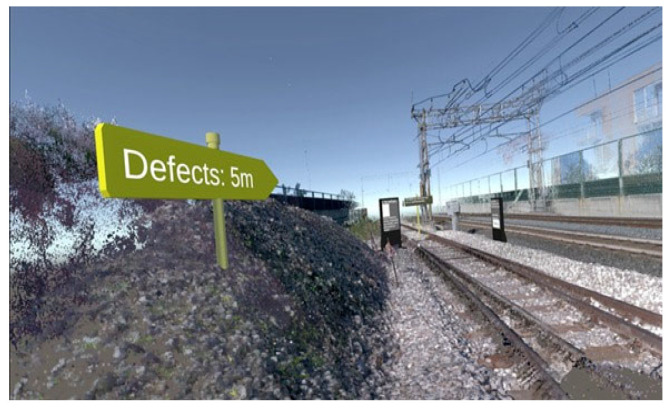
Full scene.

**Figure 3:**
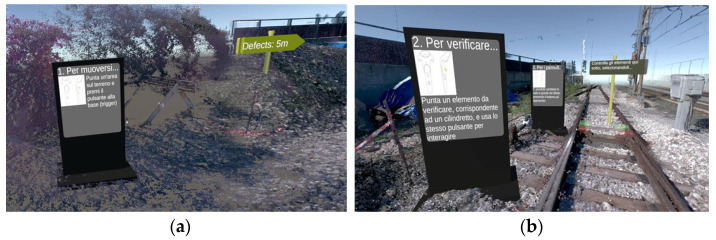
(**a**) Totem 1 instructions and (**b**) Totem 2 and Totem 3.

**Figure 4:**
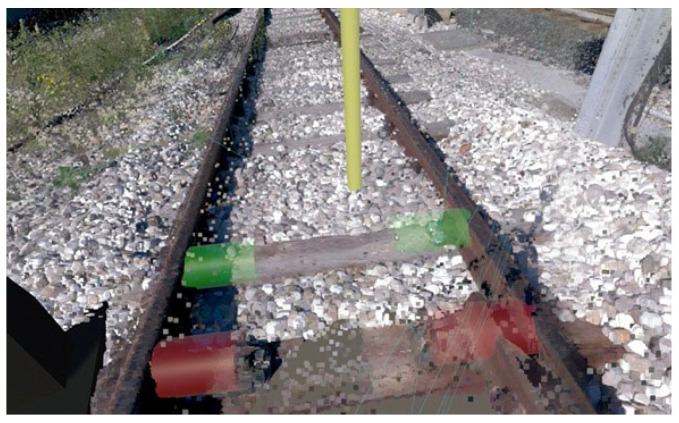
View bolts to check.

**Figure 5:**
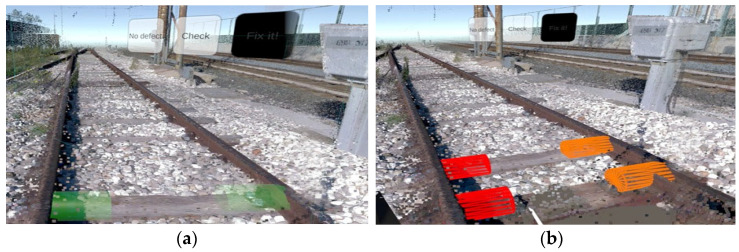
(**a**,**b**) Two different views of tabs with which to select the status of bolts.

The VR application is designed to simulate, train, or support checking and maintaining bolts on railroad tracks. Below is a complete description of its operation, highlighting its purpose and operational mechanisms:

1. Initial immersion and orientation. At launch, operators are placed in a virtual environment that mimics a section of track (see Figure 2). Users can physically “rotate” and observe the surrounding area to familiarize themselves with the environment. Virtual signs and markers (such as “Defects: 5 m”) indicate the distance and direction of the defects to be analyzed.
2. Navigation by teleportation. The application guides users through interactive “totems,” virtual structures that provide textual and graphical instructions (see Figure 3a,b). The first totem explains how to move around the environment: the user points the controller at an area of the ground and presses the basic button (i.e., trigger) to “teleport” to the desired location. On the other hand, the second and third totems illustrate the steps for checking and controlling bolts, with instructions on selecting and interacting with an element.
3. Identification and selection of bolts. Once the bolts to be checked are reached, graphical highlights (colored columns or cylinders) are noted to overlay interactive information on the track. The user, via the controller, can point to each bolt and activate contextual menus (See Figure 4).
4. Bolt status classification (see Figure 5a,b). Upon clicking on a single bolt, three representative status options appear:
  ○ No defect.
  ○ Check (to be checked).
  ○ Fix it! (needs replacement or repair).

Each time the operator selects one of these items, the cylinder associated with the bolt changes color (e.g., green, orange, or red) depending on the selected status, providing immediate visual feedback. It is worth pointing out that the classification panel can show actual pictures of the bolt or asset to be repaired, acquired by other means, such as a diagnostics train, providing a way for planners to confirm the necessity to proceed with the repair work.

**Figure 6:**
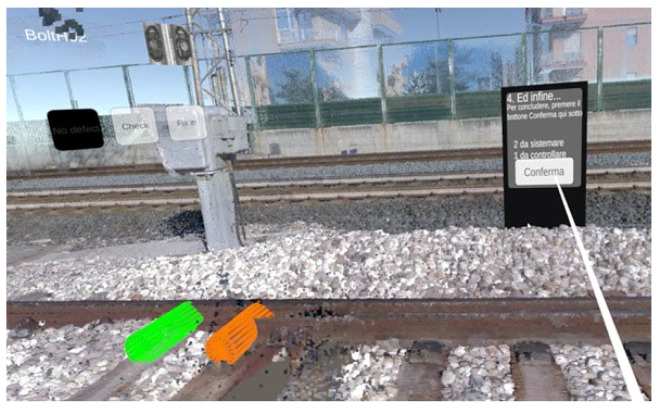
Last totem view and confirmation of activities.

5. Confirmation and completion of operations. When finished, when all bolts have been evaluated and have taken on the correct coloring corresponding to their status, the user heads to the last totem. Here, he finds the final instructions to confirm the inspection’s outcome. Once the confirmation button is pressed, the application registers that the operation is complete (see Figure 6).

With VR integration, it is possible to achieve increased accuracy in defect detection, faster maintenance processes, and better overall quality of work performed. In summary, the VR application allows:

- To train personnel in the bolt assessment and maintenance procedure in a safe and infinitely replicable context without the need to be physically present along the tracks.
- To assist operators in real time by indicating components to be inspected and actions to be taken, also in a future advanced version integrated with real systems.
- To reduce errors through a graphical UI and step-by-step instructions that minimize the risks of incorrect assessment or incomplete actions.
- To document and analyze data on bolt status (i.e., correct, to be checked, to be replaced) so that it can be tracked automatically, simplifying maintenance reports and statistics.

Concerning the previous point, it is essential to emphasize that defect assessment is primarily based on analyzing the image displayed in the panel (a bolt, in this case), rather than examining the point cloud itself. This approach is justified by two main reasons: (a) point clouds are rarely updated, whereas georeferenced images are acquired during every pass of the diagnostic train; and (b) images offer higher resolution and benefit from controlled lighting conditions, as they are captured using dedicated imaging equipment.

Conversely, the point cloud serves a complementary purpose by providing planners with a comprehensive spatial understanding of the surrounding environment. It enables them to assess whether the maintenance operation will occur in a challenging area, considering various contextual factors. For example, the terrain might be uneven; access could be limited due to the distance from entry points such as level crossings, or visibility may be obstructed by vegetation, railway curvature, and similar obstacles. These spatial insights support more effective maintenance planning by ensuring operators have the appropriate tools, resources, and scheduling.

Building upon the existing literature, our study offers a distinct contribution by introducing a highly realistic representation of railway environments within the VR module, aimed at enhancing user immersion and contextual accuracy during training and operational simulations. Unlike previous approaches that often focus on isolated systems or controlled laboratory settings, our VR solution is designed to be integrated with an AR module as part of a broader XR framework. This integration supports continuity across training and field activities, promoting consistency in how information is perceived and acted upon. Moreover, the system emphasizes user situational awareness, enabling planners and operators to engage with dynamically changing scenarios that mirror real-world complexities.

Overall, this application solution in VR is a powerful tool for assisted maintenance and training, providing an immersive, interactive experience that increases the safety, accuracy, and tracking of control operations on railroad bolts.

### 4.2. AR Application

We used Unity 2022 for developing the AR application, the Mixed Reality ToolKit (MTRK3) [26] to interface with the HMD and the Universal Rendering Pipeline (URP) for rendering tasks. The UI was implemented using MRTK3, leveraging its standard components and interaction models. The control panel appears on the user’s palm, enabling interaction through intuitive hand gestures such as the “pinch” action, ensuring a natural and accessible user experience. The system guides the user by displaying the object to be found (using a model target), and, upon recognition, overlays interactive panels that allow users to check or change the state of physical components (e.g., bolts). Visual indicators like arrows and audio cues were also integrated to help users locate and interact with elements effectively [27,28]. These features were designed to minimize cognitive load and maximize ease of use, aligning with best practices in AR usability [29]. The application was designed to run on Microsoft HoloLens 2, a mixed-reality HMD equipped with advanced spatial tracking and gesture-based interfaces. It allows users to visualize and interact with virtual objects overlaid in the physical environment. The combination of Unity 3D and HoloLens 2 enabled the development of an immersive and interactive experience, leveraging the device’s real-time rendering capabilities and environmental recognition. The AR application is designed to support operators in railroad track maintenance, particularly in identifying and resolving bolt-related defects.

The primary objective of the maintenance team is to carry out physical repairs on the railway infrastructure. Therefore, the AR solution must be designed to support their activities efficiently and unobtrusively. The VRAIL project addresses this requirement by enabling: (1) faster identification of areas requiring inspection and repair, and (2) the ability to record videos in which the operator can verbally report the observed issues. Devices such as the Microsoft HoloLens 2, combined with natural gesture-based interaction, enhance the effectiveness of this process [30]. In particular, the identification of repair locations is guided by visual landmarks that are easier to detect and are spatially close to the actual components needing inspection. These landmarks serve as initial reference points in a stepwise localization approach. Railway infrastructure elements such as level crossings and switches are suitable for this role, as they are larger and more distinctive than smaller components like bolts. The AR system and the operator then use the landmark as a spatial anchor to accurately locate the specific area requiring intervention.

**Figure 7:**
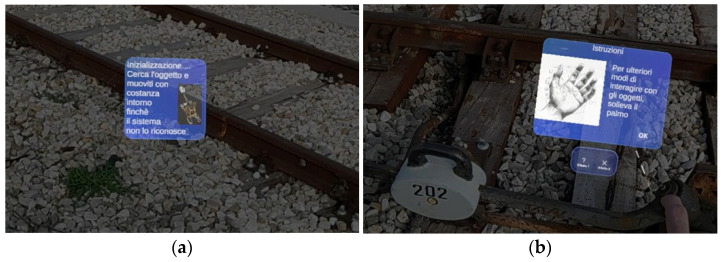
(**a**) Target object to be detected in the scene and (**b**) Instructions tab.

**Figure 8:**
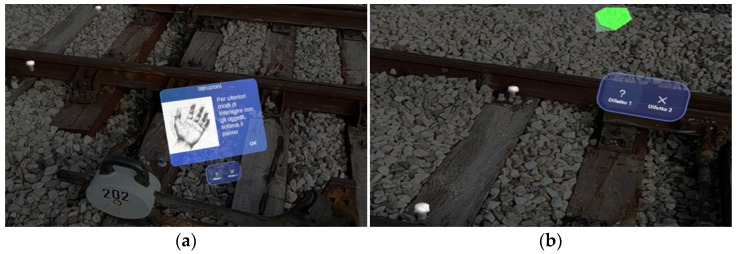
(**a**) Bolt visualization and (**b**) Arrow visualization.

**Figure 9:**
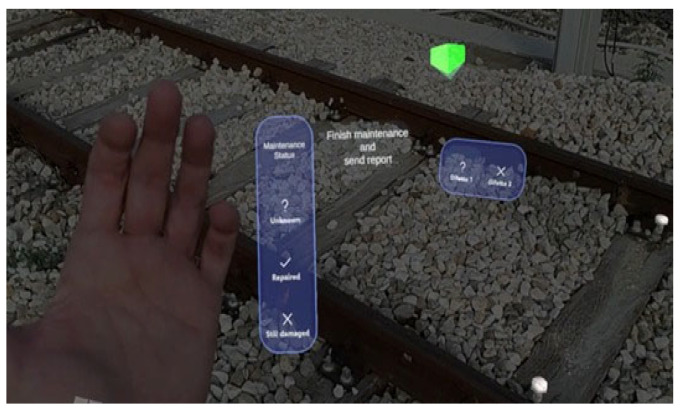
Full AR scene.

We give below a concise but rigorous description of its various functions and what they serve:

1. Target recognition and location. When the application is launched, the system shows the user a window depicting the “target object” to be searched for and framed (see Figure 7a). This “initialization” phase is used so that the head-mounted display (e.g., HoloLens or a similar device) can accurately recognize the rail component of interest (such as the joint or specific bolts on the tracks). Users moving around the target give the system different viewpoints for correct identification.
2. Instructions and AR information visualization. Once the target is recognized, a virtual interface (see Figure 7b) appears to provide operational instructions. This UI section explains how the user can interact with objects, for example, by raising a hand to bring up a menu or selecting specific options. The idea is to keep the operator’s hands free while key information appears as graphical “overlays” of the physical components.
3. Identification of defective bolts. After reading and confirming the instructions (see Figure 8a), the application directly highlights the bolts that need to be worked on in the field of view. Through visual cues (i.e., arrows, icons, or colored symbols) and spatialized audio cues, the system marks the precise points that require maintenance. In this way, the operator can immediately identify the intervention points without consulting paper documents or external maps.
4. Defect type selection. The UI then allows the user to classify the detected defects into different categories (see Figure 8b, “Defect 1” and “Defect 2”), each indicated by a symbol. In this scenario, “?” (i.e., question mark) represents the bolt to be checked, and “X” is the bolt to be replaced. By virtually touching either option, the operator indicates what intervention will be performed at the marked bolt in the application.
5. Performing maintenance. Once the defect selection is finished, the user can proceed with the maintenance (i.e., tighten or replace the bolt, as needed). This part takes place in the real world, but the AR head-mounted display (HMD) provides constant feedback on the component being serviced because of its visual overlays.
6. Maintenance status confirmation and registration. After performing operations, users lift their hands to display a new status “tab” (see Figure 9). They can confirm whether the bolts have been repaired, replaced, or remain defective. In addition, recording a video/report can be triggered so that the operator can vocally and visually document the activity performed, generating helpful tracking for later analysis or sharing the work done.
7. Process conclusion. At the end of the recording or after saving the status of all components, the operator can close the application. In this way, the intervention is “archived,” and the information collected (e.g., types of defects, corrective actions, documentation videos) can be sent to the reporting or maintenance management system.

With AR integration, it is possible to achieve increased accuracy in detecting defects, faster maintenance processes, and better overall quality of work performed. In summary, the AR application allows:

- To guide the operator step by step, showing which rail components require maintenance thanks to AR elements superimposed on reality.
- To provide instructions directly in the field of view, reducing the need to consult external manuals.
- To classify and document defects, facilitating tracking and reporting.
- To simplify communication between the operational (i.e., maintenance) phase and the back-end systems that manage interventions.

Unlike previous studies, our work introduces a novel AR approach designed for hands-free field operation, enabling technicians to interact with the system using HMDs. This design allows for precise spatial alignment of virtual elements with physical infrastructure targets, ensuring that information is contextually anchored and immediately relevant. Moreover, the system supports real-time dynamic interaction between multiple operators, facilitating effective communication and coordination during maintenance or inspection tasks. One of the key innovations lies in the ability to record operational procedures seamlessly, using visual cues and annotations that make the recorded content easily interpretable and traceable for future reference. These features collectively distinguish our AR module as a practical and field-ready solution that addresses usability and operational traceability in a way that existing AR implementations in the railway domain have yet to achieve.

## 5. Conclusions

This study is framed within the VRAIL research project and focused on the design and development of AR and VR applications to support railway infrastructure maintenance and safety. By integrating immersive visualization and interactive guidance, these technologies could offer a more effective and intuitive approach to monitoring, diagnosing, and addressing railway defects. The AR application, deployed on Microsoft HoloLens 2, enhances real-time maintenance activities by overlaying digital information onto the physical environment, providing step-by-step guidance, and streamlining defect detection. Meanwhile, the VR application developed using Unity 3D and experienced through the HTC Vive Pro 2 could offer a realistic, controlled training environment where operators can practice maintenance procedures and assess railway components without physical constraints or safety risks. The early results cited in our previous paper [4] suggest that these technologies could significantly improve efficiency, reduce operational costs, and minimize human errors in railway maintenance. Integrating AI-based anomaly detection further strengthens the framework by automating defect identification and enhancing operator decision support.

Further exploration of alternative visualization techniques may prove valuable. While anecdotal feedback suggested that the current point cloud representation was adequate, with several testers familiar with the location describing the experience as “like being there,” other forms of representation, such as polygonal meshes, may offer additional benefits. These include reduced memory usage and greater flexibility for simulating maintenance activities in more realistic scenarios. For example, alternative lighting conditions could be rendered to visualize the environment during nighttime operations.

However, implementing such features will likely require additional work in related areas. Accurately depicting a nighttime scene may involve segmenting the point cloud into distinct foreground and background elements, assigning appropriate materials and textures to each, and automatically generating collider objects. To efficiently manage these tasks, AI techniques are expected to play a key role in automating parts of the workflow.

At the project’s conclusion, qualitative feedback from domain experts indicated promising initial benefits regarding usability, intuitiveness, and perceived efficiency. However, a rigorous quantitative evaluation of performance improvements remains pending. Recognizing this, we have planned future work involving structured comparative studies, where expert and novice users will complete benchmark maintenance tasks using both conventional and immersive systems. Metrics such as task time, error rates, workload (e.g., via NASA-TLX), and traceability will be collected to evaluate effectiveness objectively. These studies are essential to confirm the observed benefits and establish the system’s scalability and generalizability.

Building on these findings, future developments will expand the system’s functionality to support additional railway defect types and integrate predictive maintenance strategies. Furthermore, efforts will be directed toward enhancing usability and accessibility to accommodate non-expert users and improve adoption in real-world settings. A key advantage of the AR application is that it allows on-field testing in real operational conditions, enabling the assessment of environmental challenges such as variable lighting and limited visibility, which can significantly impact usability and accuracy. The system’s ability to recognize physical targets and accurately anchor digital objects in the real-world scene was verified during live field tests, demonstrating its robustness and practical applicability in operational railway environments.

In parallel, VR environments present promising opportunities for future research. Specifically, virtual scenarios could be dynamically updated in real time to reflect the actual and evolving state of railway infrastructure, enabling operators and trainees to engage in continuously changing conditions. This enhancement would further increase realism, improve situational awareness, and support more adaptive and data-driven approaches to simulation and training. Finally, large-scale validation tests will be conducted to assess the long-term effectiveness and scalability of the system across different railway infrastructures. By advancing the use of VR, AR, and AI in railway safety protocols, this research paves the way for more innovative, intelligent, and efficient infrastructure monitoring practices, ensuring higher safety standards and optimized maintenance operations in the railway sector.

## Data Availability

The original contributions presented in this study are included in the article. Further inquiries can be directed to the corresponding author.
